# Perspectives of health care workers and village health volunteers on community-based Integrated Management of Childhood Illness in Madagascar

**DOI:** 10.1016/j.heliyon.2020.e05326

**Published:** 2020-11-13

**Authors:** Tomomi Kitamura, Pamela Fergusson, Arison Nirina Ravalomanda, Florentine Soanarenina, Angeline Thérése Raveloarivony, René Rasamoelisolonjatovo, Raymond Rakotoarimanana, Mitsuaki Matsui

**Affiliations:** a Bureau of International Health Cooperation, National Center for Global Health and Medicine, 1-21-1 Toyama, Shinjuku, Tokyo 162-8655, Japan; b School of Nutrition, Ryerson University, 350 Victoria Street, Toronto, Ontario, M5B 2K3, Canada; c Direction Régionale de la Santé dans le Boeny, Ministère de la Santé Publique, Complexe de la santé du Mahabibo, Mahajanga 401, Madagascar; d Bureau du District Sanitaire de Mahajanga II, Ministère de la Santé Publique, Complexe de la santé du Mahabibo, Mahajanga 401, Madagascar; e School of Tropical Medicine and Global Health, Nagasaki University, Sakamoto 1-12-4, Nagasaki, 852-8523, Japan

**Keywords:** Public health, Epidemiology, Pediatrics, Social sciences, Integrated management of childhood illness, Public health officer, Village health volunteer

## Abstract

The Ministry of Health and Family Planning of Madagascar introduced Integrated Management of Childhood Illness (IMCI) strategy in 2006, and community-based IMCI (c-IMCI), in Mahajanga II District in 2007. Following the 2009 political crisis, foreign organisations' suspension of development aid until 2012 significantly affected the implementation of c-IMCI. This study aimed to elucidate the perspectives of village health volunteers (VHVs) and public health officers (PHO) on c-IMCI. Semi-structured in-depth interviews with all VHVs working in three communes and PHOs working at central, district, and health centre levels were conducted in 2013. Textual data, created from transcripts, were translated into English and French. Data management involved analysis of sections of translated transcripts, which were marked, coded, and linked with similar experiences, challenges, and opinions; these were categorised into words and phrases to discover meaningful relationships between emerging themes. From all interviews of 30 VHV in three Mahajanga II communes and 4 PHOs, 3 themes emerged: 1) benefits of c-IMCI to the community and for VHVs, 2) challenges to continue c-IMCI, and 3) motivation to continue c-IMCI. Although all respondents considered c-IMCI as beneficial, they stated it was difficult to continue. The health system and implementation of c-IMCI should be strengthened to enable programme survival beyond the initial phase, especially during times of political instability.

## 1 Introduction

Under-five mortality in 81 countries with the highest burden of maternal, neonatal, and child mortality, including Madagascar, fell rapidly from 2000 to 2015. However, to achieve the under-five mortality rate set forth by the Sustainable Developmental Goals, of 25 per 1000 livebirths by 2030, the average annual rate of decline must be accelerated (Countdown to 2030 Collaboration 2018). In 2016, the under-five mortality rate in Madagascar was 46 per 1000 livebirths; causes of death reported in 2015 included pneumonia (15%), diarrhoea (9%), and malaria (2%) (UNICEF 2017). To contribute towards reducing mortality and morbidity in children aged <5 years in resource-poor settings, the World Health Organisation (WHO) and the United Nations Children's Fund (UNICEF) introduced an evidence-based strategy known as Integrated Management of Childhood Illness (IMCI) (WHO 2004) (Horwood et al., 2009) (Basaleem and Amin, 2011) (Febir et al., 2015) (Goga and Muhe 2011). IMCI has three components: 1) to improve case management skills of first level health workers, 2) to strengthen the health system for effective management of sick children, and 3) to promote good family and community child care practices (WHO 2004) (Horwood et al., 2009) (Basaleem and Amin, 2011) (Goga and Muhe 2011) (Pradhan et al., 2013).

The third component of IMCI, community-based IMCI (c-IMCI), can be provided through facility-based health provider outreach or at the community level by community health workers or volunteers (WHO 2004) (Basaleem and Amin, 2011) (Ghimire et al., 2010). This component focuses on improving evidenced-based key family practices to provide good home care for children (WHO 2004) (CORE Group 2009) (CORE Group). Training of health care workers (HCWs) in c-IMCI is conducted using a structured training course developed by the WHO, with at least one follow-up visit to the HCWs' health facility to reinforce their skills and solve implementation problems (Horwood et al., 2009). IMCI evaluation in five countries (e.g., Bangladesh, Brazil, Peru, Tanzania, and Uganda) showed improvements in HCW performance, increased proportion of appropriate treatment, and cost effectiveness in some settings (Horwood et al., 2009) (Basaleem and Amin, 2011) (Mitchell et al., 2013). When the protocol is followed correctly, IMCI has the potential to reduce under-five mortality (Mitchell et al., 2013).

In Madagascar, c-IMCI was introduced in 2007 in seven communes of Mahajanga II District of Boeny Region, as a pilot site (Ministère de la Santé et du Planning Familial et de la Protéction Sociale, 2007, Ministère de la Santé et du Planning Familial et de la Protéction Sociale, 2007) (Matsui and Iwamoto, 2011) (Rasamoelisolonjatovo 2014). The c-IMCI strategy targets children aged 2 months to 5 years, and addresses the care and activities provided by village health volunteers (VHVs) (see Table 1 for VHVs' activities as defined by the Ministry). VHVs were provided with medicines (cotrimoxazole, zinc, oral rehydration solution, paracetamol, artemisinin-based combination therapies [ACTs]), and equipment for their activities (Ministère de la Santé et du Planning Familial et de la Protéction Sociale, 2007, Ministère de la Santé et du Planning Familial et de la Protéction Sociale, 2007). In 2008, one year after the initial c-IMCI implementation in Mahajanga II, only 18 VHVs based in 10 sites across four communes remained active from an initial 48 VHVs based in 24 sites of Madagascar's seven communes (Matsui and Iwamoto, 2011) (Rasamoelisolonjatovo 2014). Failure to maintain c-IMCI activities, as evaluated by face-to-face interviews, was due to: 1) insufficient community support, 2) no supervision from higher levels (health centre or district health office), 3) no continuous supply of medicine and equipment, and 4) personal reasons (for example, moving away) (Matsui and Iwamoto, 2011) (Rasamoelisolonjatovo 2014). Conversely, reasons for continuing the activities were: 1) head of the health centre actively collaborating with and facilitating communication with local authorities, 2) support from the local authorities to implement c-IMCI activities, and 3) the community constructing c-IMCI sites (Rasamoelisolonjatovo 2014). Based on these findings, the District Health Office of Mahajanga II reactivated c-IMCI in 2009 through advocacy, training, and supervision (Rasamoelisolonjatovo 2014). However, a political crisis affected Madagascar in 2009, resulting in foreign organisations' suspension of development aid due to the absence of democratic progress (BBC 2015). This jeopardised c-IMCI, and VHVs found it difficult to continue their activities. In 2011, a signed agreement by political parties to pave the way for elections within a year led financial support provided by the Global Fund for the national programme against malaria. In June 2012, part of the budget was to be allocated to c-IMCI and relevant training. This study aimed to explore the perspectives of VHVs and public health officers (PHOs) involved in c-IMCI in Mahajanga II after the second attempt at c-iMCI implementation reactivation during the political crisis.

**Table 1:** Community-based Integrated Management of Childhood Illness: Care practices and activities provided by the village health volunteers.

Weigh the sick children
Care for acute respiratory infection, malaria, and diarrhoea
Check the child's immunisation, vitamin A, and deworming status
Refer the child with any general danger sign to the health centres
children Set a follow-up visit and give follow-up care
Management of medicine supplies
Report community Integrated Management of Childhood Illness (c-IMCI) activities (diagnosis, treatment, follow-up and referral) to the health centres
Organise Information, Education, Communication (IEC) sessions and social mobilisation
Promote 18 key family practices∗ set by the Government of Madagascar

∗Promotion of growth and development

- Breastfeed children exclusively during the first 6 months

- Introduce complementary foods from the second 6 months and continue to breastfeed up to 2 years or longer

- Ensure that children receive adequate micronutrients - such as vitamin A, iron and zinc - in their diet or through supplements

- Promote mental and social development by responding to a child's needs for care and by playing, talking and providing a stimulating environment

- Ensure regular follow-up and promotion of growth until a child reaches five years of age

Disease prevention

- Dispose of faeces safely, wash hands after defecation, before preparing meals and before feeding children

- Protect children in malaria-endemic areas by ensuring that they sleep under insecticide-treated bed nets.

- Provide appropriate care for HIV/AIDS-affected people, especially orphans, and take action to prevent further HIV infections.

- Ensure prevention of dental caries by appropriate cares for teeth.

Appropriate care at home

- Continue to feed and offer more fluids, including breastmilk to children when they are sick.

- Give sick children appropriate home treatment for infections

- Protect children from injury and accident and provide treatment when necessary

- Protect child abuse and neglect, and take action when it does occur - Involve father in the care of their children and in the reproductive health of the family

Care-seeking outside the home

- Recognise when sick children need treatment outside the home and seek care from appropriate providers

- Take children to complete a full course of immunisation before their first birthday

- Follow the health provider's advice on treatment, follow-up and referral

- Ensure that every pregnant woman has adequate antenatal care and seeks care at the time of delivery and afterward.

## 2 Materials and methods

### 2.1 Setting

**Figure 1:**
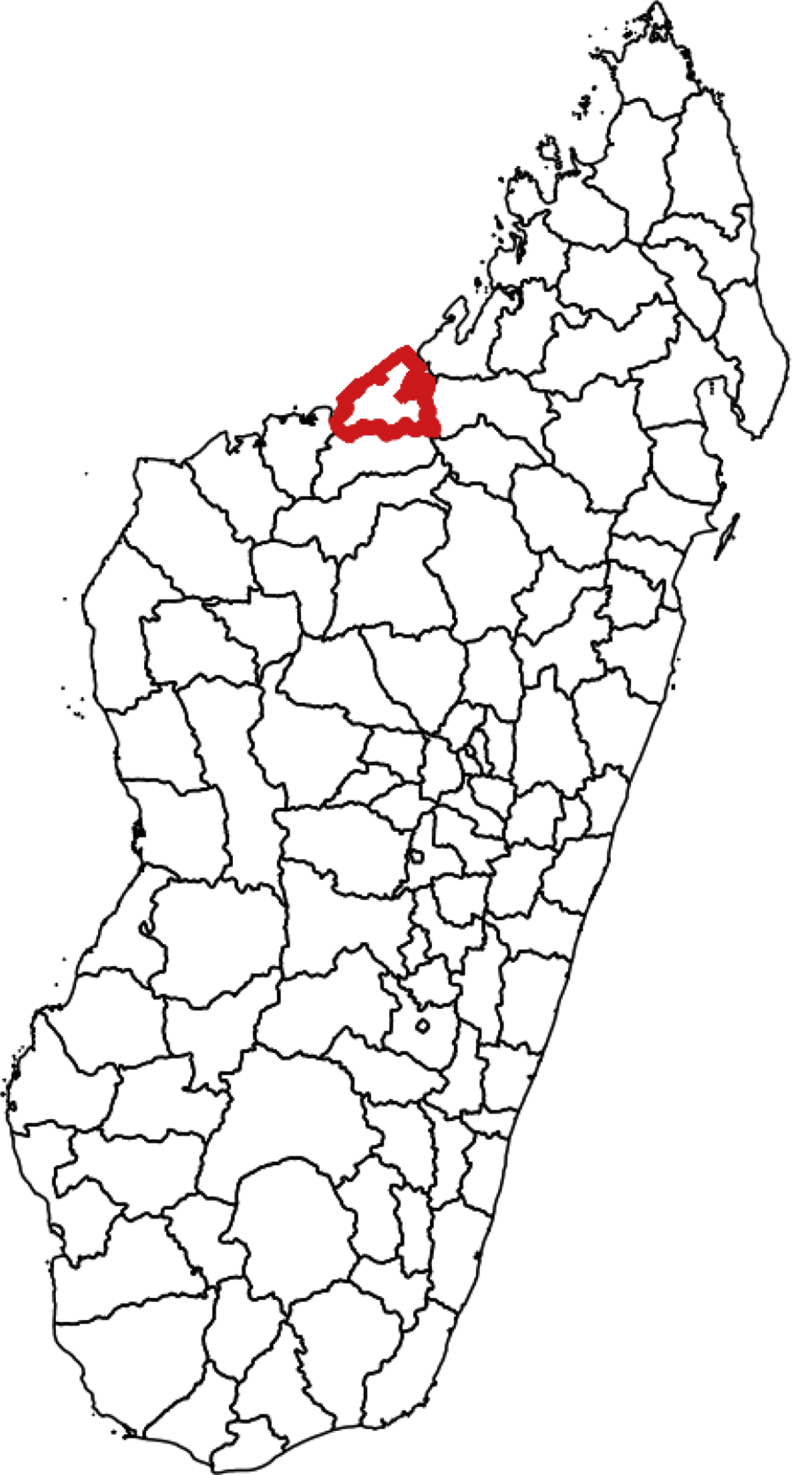
Mahajanga II District. Mahajanga II District is in the north-west region of Madagascar.

Mahajanga II District, located in the north-west of Madagascar, covers an area of 4,568 km^2^ with a 2011 population of 60,515 (Figure 1). The district consists of nine communes, each with an average of eight villages (with 75 total villages in Mahajanga II). In 2012, 19 health centres, representing the first level health facilities, covered the district. As a result of limited access to the health centres, the c-IMCI programme was implemented in 2013 in nine out of ten villages, three out of nine villages, and four out of five villages in the three communes (Bekobay, Belobaka, and Betsako, respectively) targeted by this study.

### 2.2 Respondents

This study included all VHVs (n = 30) working in the three communes in Mahajanga II at the time of interview in July 2013. PHOs (n = 4), one each at central and district levels, and two at the health centre level, were also interviewed.

### 2.3 Data collection

The study was conducted in July 2013, which was six years after the initial training of c-IMCI and a year after a part of the budget was to be allocated to c-IMCI and relevant training. Data collection involved semi-structured in-depth interviews. The researcher interviewed the respondents using an interview guide written in English and French and translated into the local language (Malagasy). The interviews were audiotaped and transcribed. Any relevant remarks, including facial expression of the interviewees, were recorded in field notes (Basaleem and Amin, 2011).

### 2.4 Data analysis

Textual data were created from the transcripts and translated from Malagasy into English and French by a local translator who did not participate in conducting interviews, to ensure data credibility and dependability. Data management and analysis of interview transcripts was performed using Nvivo 8 (QSR International Pty Ltd, Australia) (Febir et al., 2015). Sections of translated transcripts, marked and coded by the researcher and linked with similar experiences, challenges, and opinions, were categorised into words and phrases to discover meaningful relationships between emerging themes. Emerging themes with significant overlaps or conflicts were checked for possible relationship to the data; these transcripts continued to be reviewed and refined.

### 2.5 Ethical considerations

This study was approved by the ethical committee in Madagascar (Conseil Régional de l’Ordre des Médecins de Boeny; reference number: 01.07 PDT.CRB 2012) and Japan (National Centre for Global Health; reference number: 1018). Written consents were obtained from all participants prior to the interviews and all the participants received an explanation of the objective of the research, confidentiality of collected data, and participant rights to withdraw from the study. On the day of the interview, all respondents received a daily allowance according to an agreement between the Ministry of Health in Madagascar and the National Center for Global Health and Medicine in Japan.

## 3 Results

From the 22 in-depth interviews, involving 30 VHVs and 4 PHOs, three themes emerged: ‘benefits of c-IMCI,’ ‘challenges to continue c-IMCI,’ and ‘motivation to continue c-IMCI’ (Table 2).

**Table 2:** Perspectives of village health volunteers and public health officers on c-Integrated Management of Childhood Illnesses.

Themes	Codes from village health volunteers	Codes from public health officers
Benefits of c-IMCI	**Benefits for the community**	**Benefits for the community**
	-Health care nearby	-Health care nearby
	-Progress in children's health	-Progress in children's health
	-Cheap health care	
	-Prevention	
	-Sensitisation	
	-Smooth referral	
	**Benefits for the village health volunteers**	**Benefits for the village health volunteers**
	-Knowledge	-Knowledge
	-Skills	
	-Treatment for his/her own children by him/herself	
	-Compensations (e.g. rice)	-Compensations (e.g. Selling medicine)
		-Recognition
		**Benefits for the Ministry of Health**
		-Tackling high infant mortality rate
Challenges to continue c-IMCI	-Weak system on management	
	-Unstable/weak support	
	Management	-Unstable support
		Management/Financial
	-Collaboration	-Political situation
	-Medicine supply	-Collaboration
	-Equipment supply	-Medicine supply (starting lot)
	-Site for c-IMCI	-Equipment supply
	-Supervision	
	-Training	-Supervision
		-Training
Motivation to continue c-IMCI	-Feeling this is for children/good action for children	
		-Sense of responsibility
		-Recognition
		-Passion
	**Ideal motivation**	**Ideal motivation**
	-Compensation	
	-Contract	-Contract

### 3.1 Benefits of community-based Integrated Management of Childhood Illness

Overall, all respondents considered c-IMCI as beneficial both to the community and to VHVs.

### 3.2 Benefits of community-based Integrated Management of Childhood Illness to the community

The respondents stated that c-IMCI benefited the community because it improved the children's health by enhancing access to health care nearby, other than heath facilities.

<VHVs>

*‘Their advantage is not to move far when their children are ill, we (the VHVs) are nearer.’*

<VHVs>

*‘In our village since this c-IMCI has been here, since it has been set up, as for the children first here with us, there weren't any dead children even for these questions of malnutrition.’*

Furthermore, the VHVs described other benefits related to c-IMCI activities, such as low cost, access to information on preventive measures regarding childhood diseases, sensitisation on child care, and smooth referral to the health centres. Health care was considered low cost because VHVs' medicines were cheaper than those sold either at the market or at the health centre. Referrals were considered ‘smooth’ because in two health centres, c-IMCI involvement was required before they were accepted.

### 3.3 Benefits of community-based Integrated Management of Childhood Illness for village health volunteers

All respondents mentioned benefits to the VHVs. The most valued benefit was the knowledge gained through c-IMCI training.

<VHVs>

*‘The advantages for us, our knowledge is really extended. And as we didn't succeed at school, these are advantages. As we've got children, they won't have any difficulties when they are ill.’*

### 3.4 Challenges to continue community-based Integrated Management of Childhood Illness

Several challenges affected continuation of c-IMCI by VHVs. Some of these factors were managerial or systematic challenges from the central to the community level, while others were at the community level.

<VHVs>

*‘The problem posed following the c-IMCI is a problem coming really from upper level there. For example, concerning the NGO (who supports the c-IMCI), the NGO always changes then. That's what we really noticed, that it often changes then and its organiser then creates some problems for us'.*

<PHOs>

*‘As you (the international organizations) were supporting us for 5 years, those times passed, they are leaving and we, we are going to stay…hum…hum. We can't do anything afterwards. Sustainability is also very important to be able to work, always to continue the work.’*

<PHOs>

*‘I think it's difficult to continue. It's perhaps easy to begin, but it's difficult to continue. However, all the same, we try to survive.’*

### 3.5 Collaboration

Both VHVs and public health officers considered effective collaboration between VHVs, village heads, and health centres to be a key challenge for c-IMCI continuity, but that this would be a facilitating factor once it worked.

<VHVs>

*‘Collaboration with head of village is important, otherwise it is very difficult.’*

<VHVs>

*‘When we are all in a meeting, we see if this doesn't go well, what you don't understand about this, then he (head of health centre) explains. He corrects and you ask questions, then he explains. Even if you don't ask any questions he always knows what you do not understand: he explains, here is what can't go with this; this is like that, and if there is something you don't understand, he explains to us.’*

<PHOs>

*‘Me, I haven't got any problems with the ACs (VHVs). But with the local authorities and the sites, it's difficult. Very difficult to dissuade them.’*

### 3.6 Medicine supply

VHVs were supposed to be provided with medicines after their training, and the sale of medicines was the compensation for their work. However, there was either delay or no provision of the medicines. Although the medicines were available for some of the VHVs at the time of the interview, VHVs expressed their discontentment about irregular sales of medicines, which were insufficient as compensation.

<VHVs>

*‘Now, we haven't yet received any medicine. We have never had, but it's with our own money that we buy some to be able to work.’*

<VHVs>

*‘There were times when there were not any drugs. The government was disorganised. And we have been doing (our work) even though there were no drugs. We always encourage the sick children to come. We are acting like we know something to do.’*

<PHOs>

*‘I think that if we'd like it to function well, starting, lots must be given, like for the openings of the CSBs 1 (health centres), or like that.’*

### 3.7 Equipment supply

In this study, the VHVs reported several missing items needed for their work, such as chairs and tables, watches or timers, and scales. Some of the VHVs stated that their bicycles did not function well in their communities due to the road conditions or the long distance between the health centres and their communities. The VHVs also mentioned that they ran out of forms or needed to photocopy the forms themselves, which involved going to town.

<VHVs>

*‘We need chairs, tables, watches for the work for we haven't got any, because they introduced the watch within our training.’*

<VHVs>

*We wish to have a bike. It's speedier. And with a motorbike, the two of us can go, and then we go there.*

<PHOs>

*‘The bikes are not too functional because there are some missing parts and there it's still another problem because Marofiatsaka and Ampampamena are very far. The bikes are not very practical. Even to come here (health centre) to bring the report.’*

<VHVs>

*‘We still have the management forms, however, we do not have the referral form and the report form to the CSB. That's our problem. The main problem is that we have to go to Mahajanga to make the photocopies.’*

### 3.8 Site for consultations

In Madagascar, the VHVs expected the community to build a site for c-IMCI consultation and for storing medicines and equipment instead of receiving the children at their homes. This study revealed positive support from the head of village or the community to build the site. However, some communities were still unable to provide the site, and in those cases the VHVs felt that the community was not supportive enough. In the absence of c-IMCI sites, the VHVs used their homes; however, this resulted in worry that the medicines might not be properly stored, or worry over the possibility of nosocomial infections between patients and VHVs' family members.

<VHVs>

*‘The problem is that we do not have any site to work in, but we work at home’.*

<VHVs>

*‘Then as we are in the bush, there is a lot of dust. And everything is mixed, when I clean and do the sweeping of the house, the dust damages the medicines.’*

<VHVs>

*‘There are babies there (at home). For the babies then, we are afraid that they will get infected when I receive ill children’.*

### 3.9 Supervision of village health volunteers

The VHVs received irregular on-site supervision from the heads of health centres, although they had regular meetings with them at the health centres. Although the VHVs appreciated the effort of the heads of the health centres, they were not satisfied with the inadequate supervision; additionally, the supporting NGOs also failed to organise supervision at the community level.

<VHVs>

*‘We need that there are frequent supervisions for us here, because sometimes there is then something which doesn't go well, either it's a question of forms, fee for the child or a question of child reception then. That's what it is really about it. We need help.’*

<VHVs>

*‘They (supporting NGO) said they would come here but they didn't come here. They only came to the CSB (health centre) there, and went back home.’*

<PHOs>

*‘Generally, there is no benefit for me (to do the supervision). It's just to give my agents some advice to motivate them, or what. But for me, I've got no benefit, but even to go there with my motorbike, I spend out of my own pocket, the fuel.’*

### 3.10 Training of village health volunteers

Many of the VHVs in this study were trained more than once, as c-IMCI was implemented in 2008 and reintroduced in 2009. The c-IMCI training was considered well-organised and easy to understand. PHOs were concerned that no refresher training had taken place to maintain the quality of the practice.

<VHVs>

*‘Our training was easy enough to be understood, it wasn't too difficult.’*

<VHVs>

*‘Even if we are from the bush here, these doctors there, you see, they teach all these things there, they don't show they are unpleasant, but when you say ‘That's what I don't know how to do, doctor’, then they teach how to. ‘Do this way, do this like that, and for this you do that way.’*

<PHOs>

*‘There, there is a refresher at the level of the people (community level) at least once a year or… There should be a refresher or grouped follow-ups and the supervision should also be done at least every six months, or if we can't go there every two or three months; at least there should be one formative supervision twice a year, shouldn't it?’*

### 3.11 Motivation to continue community-based Integrated Management of Childhood Illness

Despite the challenges described above, the VHVs fulfilled their duties because they felt that their work influenced the health of the children in their communities. Although the VHVs were diligently persevering in their work, they wished to receive some compensation for their work.

<VHVs>

*‘We are willing to do this work because it is the pillar of the health here in our village and we prefer then to really make efforts so that the fact that our children can enjoy health is fulfilled.’*

<VHVs>

*‘You can manage ill children in charge of yourself, doing good to the children then!’*

PHOs assumed that VHVs continued with their work due to a sense of responsibility, the recognition received from the community, and their passion for the work.

<PHOs>

*‘They were very interested about health because it's really vital, for they know that we (health centres) are far. So, they are conscious of their responsibility.’*

<PHOs>

*‘To be ‘doctors’ in the bush is also a prestige for them. It's the sense that it has succeeded.’*

### 3.12 Compensation

The VHVs wished to be compensated for their work or to be contracted as part of the public health sector so that they could receive a regular salary. The VHVs justified their entitlement to compensation on the following grounds: their work was hard and benefited the community, income received from selling medicine was not enough or regular, they had to leave their work at home or in the fields when ill children came, they were sending reports to the health centres, and their family complained if they did not receive any compensations.

<VHVs>

*‘The work is very hard though there is not the small compensation; it's as if it has introduced a problem in our home too. I have to leave home to work here. My husband is not happy with me going out. It's a work without money. In order to convince my husband, I can say ‘this (work) can be something that helps us’.’*

<VHVs>

*‘We would like to be really hired within the framework of the health agents there, as a part of a health staff. They earn every month, and you also get a small benefit in your pockets to develop your knowledge then with your own skills there.’*

The PHOs suggested that it may be a form of compensation if the VHVs were provided with sufficient medicines from the outset, as described above. However, the VHVs were not satisfied with this proposal as the earnings from the sale of medicines were small and irregular.

< PHOs>

*‘As for me, I think that if you give them, if you supply the starting drugs, which may help them because there is a percentage which they will earn from the medicines. So, if them, they obtain the drugs, they can work, and as they are going to work, they will get much experience.’*

<VHVs>

*‘What is about the medicines: for example, we take some medicines at 20 ariary at their place there, we fetch for them at the CSB, that is we take and buy them at 20 ariary there, then we sell them at 30 ariary here? That shouldn't be the salary which would hold us in the site.’*

The PHOs expected the community to provide some form of compensation to the VHVs. However, the VHVs and even some of the PHOs were sceptical about the sustainability of this strategy.

<PHOs>

*‘I have already talked with the leaders at the level of the communities about these motivations. Some sacks of paddy can be given. I have already given these ideas but to the sites of Betsako. They said ‘yes, we can do, yes we can do’, but up to now, I haven't heard about it. Even for Bekobay, I already talked with the head of the Fokotany (village), and like the case of Marofiatsaka, the population – these people give I don't know if it's 5 sacks of paddy per year as a motivation. And moreover, they plough the rice field. But I don't know whether they still do it now or stopped.’*

<VHVs>

*‘You (VHV) are from the population, collaborating with the head of Fokotany, there should be some contribution of the population so that you can get even a small benefit’. I transmitted the message to the population. They cannot afford to do so (to pay for the VHVs).’*

The PHOs also considered that implementing contracts with VHVs may increase their motivation.

<PHOs>

*‘The VHVs will be taken as contractual or something like that, such as the agents of the Civil Service with us; but faced to the present situation, that hasn't yet been led to, but it seems that will come. Perhaps that will motivate them much more.’*

## 4 Discussion

This study discovered that VHVs and PHOs in Madagascar considered c-IMCI as beneficial both to the community and to VHVs themselves. However, both the VHVs and the PHOs stated that, despite being motivated, it was challenging to continue c-IMCI. Furthermore, they provided some suggestions for creating the ideal motivation to continue with c-IMCI.

### 4.1 Benefits of community-based Integrated Management of Childhood Illness

The benefits reported in this study are similar to those identified in previous studies. A study in Pakistan reported that most HCWs interviewed reported IMCI as important for improving children's health (Pradhan et al., 2013). One HCW, in a qualitative study in Yemen, reported that ‘People appreciate good treatment near them’ and ‘There is less malaria and severe dehydration, no deaths from severe malnutrition or diarrhoea, severe pneumonia and referral cases are fewer, and many communicable diseases have disappeared’ (Basaleem and Amin, 2011). The benefits to the providers were also comparable with previous studies. A study from South Africa reported increased confidence in managing children by HCWs, and that they felt empowered and knowledgeable in their practise (Horwood et al., 2009). Additionally, the study from Yemen showed that health providers viewed their IMCI experience as ‘great and worthy’ (Basaleem and Amin, 2011).

### 4.2 Challenges to continuing with community-based Integrated Management of Childhood Illness

Weak health systems and their related challenges exist in many developing countries, and IMCI does not always adequately address these challenges, as IMCI implementation tends to focus on the training of providers rather than on health system strengthening (Basaleem and Amin, 2011) (Febir et al., 2015). Because it is difficult to develop c-IMCI based on an existing fragile health system, it is necessary to plan beyond programme implementation to ensure continuity, particularly during a period of political instability. PHOs admitted that ‘it was easy to start but difficult to continue c-IMCI’ while VHVs reported that the unstable and weak system directly affected their work in the field. Pradhan et al. reported on concerns related to absence of a clear initial planning phase for the optimal implementation of IMCI, and, based on in-depth interviews with stakeholders of facility-based IMCI, pointed out a time lag between initial training and initiation of IMCI activities (Pradhan et al., 2013). Some respondents in this study reported a delay in the initiation of activities due to poor drug supply, which hindered the implementation of c-IMCI. Despite the difficulties, the respondents in this study persevered. In the future, the resilience of the health system should be strengthened in tandem with IMCI implementation, enabling proper planning at initial stages, building of necessary infrastructure and supply system, planning and guaranteeing a budget for training/refresher training followed by regular field supervision, adequate support, and appropriate incentives to motivate VHVs (Das et al., 2013) (Kieny et al., 2014) (WHO 2017).

One year after the initial implementation of c-IMCI in Madagascar, both VHVs and PHOs considered the collaboration between VHVs, village heads, and health centres as important, and this study identified community-level efforts to establish a close link between these stakeholders (Rasamoelisolonjatovo 2014). A Yemen study indicated that a close link between health facilities and the community was essential, highlighting the importance of defining the role of community health workers early, at the stage of approaching the community (Basaleem and Amin, 2011).

This study revealed that interviewees considered medicine supply to be an ongoing key challenge, six years after initial implementation. However, some positive improvements of the management of medicines, such as small deposits provided by the villagers to maintain the medicine stocks or preserve a well-functioning health centre pharmacy for the VHVs, were discovered by a previous study in Mahajanga II District (Rasamoelisolonjatovo 2014). The VHVs in this study also described challenges related to inadequate supply of equipment, which had been reported as one of the reasons that VHVs were unable to continue their activities in 2008 (Rasamoelisolonjatovo 2014). An effective supply system for medicines and equipment is essential to the implementation of c-IMCI. A weak supply system, according to studies from Pakistan and Tanzania, can prevent HCWs from putting their training into practice; additionally, this was one of the factors responsible for HCWs' inability to implement IMCI continuously (Pradhan et al., 2013) (Kiplagat et al., 2014). Effective service delivery requires trained and supervised staff working with appropriate medicines and equipment, adequate financing, and suitable incentives for providers and users (WHO 2007). A study from Pakistan showed that weak governance, such as ambiguous roles and responsibilities among stakeholders, can translate into a lack of supply and logistics (Pradhan et al., 2013).

To facilitate the continuity of c-IMCI in 2008, communities in Madagascar were expected to build a community-based site for c-IMCI consultation (Rasamoelisolonjatovo 2014). The need for a specific building site for IMCI has not received much attention in the literature. This may be because HCWs, rather than VHVs, have been the target of previous studies, and normally these workers have a medical facility in which to provide medical care to children and to store medicines and equipment. In a Pakistan study, to facilitate IMCI implementation, a separate under-five clinic at the primary health centre was suggested (Pradhan et al., 2013).

Lack of supervision was one of the reasons found in this study for not continuing c-IMCI, as in 2008. Supervision was often delayed, or only a few ongoing clinical supervision sessions took place due to a number of factors: lack of leadership; inadequate management; poor coordination between managers of the programme and donors; lack of funding, including travel costs for supervision; problems with decentralisation; the low priority assigned to supervision; inadequately trained supervisors; an inadequate number of trained and skilled supervisors; inadequate job aids for follow-up; lack of motivation among supervisors; and an expectation of integrated supervision (Horwood et al., 2009) (Goga and Muhe 2011) (Rowe et al., 2010). Studies from Pakistan and Tanzania showed that lack of supportive supervision or on-site mentoring were among factors that hindered IMCI implementation (Pradhan et al., 2013) (Kiplagat et al., 2014). A systematic review by Rowe et al. showed that adequacy of treatment dropped when there was no intervention after the training, and supervision appeared to be effective in improving the treatment (Rowe et al., 2012). This study revealed that motivated heads of health centres conducted irregular on-site supervision at their own expense, and they also conducted regular meetings with VHVs at the health centres (Rasamoelisolonjatovo 2014). These efforts might conceal areas of weakness in supervision or follow-up from the higher levels of the health system.

This study showed that VHVs found their training to be well-organised and easy to understand. The PHOs were concerned about quality of care due to lack of regular refresher trainings. Few VHVs expressed these concerns, which may be because many of them underwent training more than once, although training organisers did not intend the repeat training sessions to be refresher trainings (Das et al., 2013). Previous studies showed mixed results regarding the view of VHVs on IMCI training. A South Africa study on IMCI training experiences among HCWs reported that they ‘found the training interesting, informative and empowering and there was consensus that it improved their skills in managing sick children’ (Horwood et al., 2009). The same study reported that ‘HCWs also felt that repetition was important to reinforce knowledge and skills’ (Horwood et al., 2009). Conversely, the study from Tanzania found that over half of the HCWs reported that the training was inadequate because it took place in a workshop setting, and follow-up, including on-site mentoring and refresher trainings, were necessary (Kiplagat et al., 2014). A systematic review by Das et al. reported that the accuracy of treatment dropped when there was a gap between initial and refresher training sessions.

### 4.3 Motivation to continue community-based Integrated Management of Childhood Illness

In this study, the VHVs strongly believed that they should be compensated for their work. These views may have been strong because of the unpredictable and difficult social and economic situation in Madagascar at the time of the interviews. In Benin, the Ministry of Health implemented an incentive policy, together with quality improvement activities that included monthly meetings and quarterly learning sessions, and showed that this approach correlated with the quality and contribution of community volunteers to the implementation of c-IMCI (Febir et al., 2015) (USAID Maternal and Child Survival Program 2015). Das et al. (2013) also highlighted the importance of incentives when community-based HCWs work outside of the formal health system and are expected to work as volunteers; a lack of incentives was shown to have negatively affected the retention of healthcare workers. In Madagascar, the quality of c-IMCI would be improved through strengthening of regular informal supervision, promoting monthly meetings by heads of health centres, and delivering regular incentives. The challenges and strategies to promote c-IMCI continuation, as described above, were interdependent. The overarching issues appeared to be poor leadership, supervision, and collaboration; these led to inadequate initial planning and poor preparation for c-IMCI implementation and sustainability (Pradhan et al., 2013).

This study has several limitations. Firstly, it was conducted in one region of Madagascar and thus the findings may not be generalisable to other settings. Secondly, the study was unable to obtain the perspectives of VHVs who were not active in c-IMCI. Lastly, this study concentrated on the perspectives of providers and the managers of the programme and did not reflect the view of the beneficiaries.

## 5 Conclusions

This study evaluated c-IMCI in three communes of one district in Madagascar, from the perspective of VHVs responsible for implementing the programme in the field and PHOs in charge of managing the programme. The context of c-IMCI implementation in Madagascar has been shaped by repeated attempts at implementation and reactivation during a period of political instability. To the best of our knowledge, this study is the first to record the perspective of both implementers and managers of c-IMCI in Madagascar. Both VHVs and PHOs viewed c-IMCI as being beneficial to the community and to the VHVs themselves. However, this study identified some challenges against continuity of c-IMCI implementation, such as weak health systems, collaboration, supply, infrastructure, supervision, and training. This study also found that VHVs strongly believed that compensation for their work in the field was necessary to offset their time and personal expenses given towards the programme, and to continue their involvement with c-IMCI. To tackle these interrelated challenges, health systems should be strengthened to enable implementation of the IMCI programme, survival at its initial phase, and ongoing effectiveness, even during times of political instability.

## Declarations

### Author contribution statement

Tomomi Kitamura: Conceived and designed the experiments; Performed the experiments; Analyzed and interpreted the data; Contributed reagents, materials, analysis tools or data; Wrote the paper.

Pamela Fergusson: Conceived and designed the experiments; Analyzed and interpreted the data.

Arison Nirina Ravalomanda, Florentine Soanarenina, Angeline Thérése Raveloarivony, René Rasamoelisolonjatovo, Raymond Rakotoarimanana: Performed the experiments; Contributed reagents, materials, analysis tools or data.

Mitsuaki Matsui: Conceived and designed the experiments.

### Funding statement

This work was supported by The Grant for 10.13039/100012319National Center for Global Health and Medicine [grant number 23-3].

### Competing interest statement

The authors declare no conflicts of interest.

### Additional information

No additional information is available for this paper.
